# Efficacy of a modified Double-Ovsynch protocol for the enhancement of reproductive performance in Hanwoo cattle

**DOI:** 10.5713/ab.22.0353

**Published:** 2022-01-10

**Authors:** Jae Kwan Jeong, Ui Hyung Kim, Ill Hwa Kim

**Affiliations:** 1 College of Veterinary Medicine, Chungbuk National University, Cheongju 28644, Korea; 2 Hanwoo Research Institute, National Institute of Animal Science, Rural Development Administration, Pyeongchang 25340, Korea

**Keywords:** Estrus Detection, Hanwoo Cows, Modified Double-Ovsynch, Reproductive Performance, Timed Artificial Insemination

## Abstract

**Objective:**

We aimed to evaluate the efficacy of a modified Double-Ovsynch protocol vs artificial insemination following estrus detection (AIED) for the enhancement of reproductive performance in Hanwoo cattle.

**Methods:**

Four hundred twelve Hanwoo cows were allocated to two treatment groups. The first group of cows were administered gonadotropin releasing hormone (GnRH) on Day 36 (±0.6), prostaglandin F_2α_ (PGF_2α_) on Day 46 (8 to 12 days later), and GnRH on Day 49, which was followed by Ovsynch, consisting of an injection of GnRH on Day 56, PGF_2α_ on Day 63, and GnRH 56 h and timed AI (TAI) 16 h later (modified Double-Ovsynch group, n = 203). The second group of cows underwent AIED (AIED group, n = 209) and were designated as controls.

**Results:**

The pregnancy per AI 60 days after the first AI was higher in the modified Double-Ovsynch (68.5%) than in the AIED (56.5%) group, resulting in a higher probability of pregnancy per AI (odds ratio: 1.68, p<0.05). Moreover, cows in the modified Double-Ovsynch group were more likely (hazard ratio: 1.28, p<0.05) to be pregnant by 150 days after calving than cows in the AIED group, and this difference was associated with a lower mean number of AIs per conception (1.27 vs 1.39, p<0.05) and a shorter median interval between calving and pregnancy (72 vs 78 days, p<0.1).

**Conclusion:**

The modified Double-Ovsynch protocol, adjusted according to the herd visit schedule, can be readily used to increase the pregnancy per AI following the first AI and to shorten the interval between calving and pregnancy in beef herds.

## INTRODUCTION

Korean native cattle (Hanwoo, *Bos taurus coreanae*) are the most popular breed of beef cattle farmed in Korea and represent an important source of meat [1]. Recent changes in livestock production, such as increases in herd size and reductions in the size of the labor force, have made estrus detection more difficult, which has reduced the reproductive efficiency of Hanwoo cattle. In addition, prolonged postpartum anestrus is an important cause of impaired fertility in Hanwoo cows, as for other beef breeds [2,3]. Postpartum anestrous in beef cows is associated with the presence of the calf and poor nutrition [4,5], and results in lower levels of secretion of gonadotropin releasing hormone (GnRH) and luteinizing hormone (LH), which impair final follicular development and ovulation [6,7]. Prolonged postpartum anestrus of >80 days occurs in 40% of Hanwoo cows farmed by smallholders, who typically keep the cows tethered and feed them hay and rice straw [8]. A previous study showed that a mean 23% of suckled beef cows do not ovulate before attempts at breeding commence [9], and only 54% of 3,269 beef cows were shown to be cycling before the start of the breeding season [3]. Furthermore, longer postpartum anestrous periods (>100 days) were identified in *Bos indicus* beef cows kept under a continuous grazing system [10].

Overcoming the problems of prolonged postpartum an estrus and poor estrus detection is very important for the maximization of fertility in beef herds. Therefore, substantial effort has been devoted to solving these problems and thereby improving reproductive performance in suckling beef cows, in particular in the form of timed artificial insemination (TAI) protocols, because such protocols have the advantages of reducing the time and effort necessary for estrus detection. The administration of GnRH induces the ovulation of a dominant follicle and the development of a new follicular wave in cattle [11], and synchronization ovulation protocols involving the administration of GnRH and prostaglandin F_2α_ (PGF_2α_) have been used in both dairy and beef cattle production [12,13]. Ovsynch or CO-Synch programs (which comprise GnRH administration on day 0, PGF_2α_ administration on day 7, and a second GnRH injection and concurrent TAI on day 9) were previously included in synchronization ovulation protocols for beef cows, yielding pregnancy per AI of 57% and 49%, respectively [13]. Moreover, the addition of a progesterone-releasing device (a controlled internal device release, CIDR) between days 0 and 7 of the CO-Synch protocol was associated with a higher pregnancy rate (58%) than in cows that lacked a CIDR (48%) [9]. However, another study showed that the prolonged postpartum anestrus limits the efficacy of such synchronization ovulation protocols in Japanese beef cows [14].

Progestin-based synchronization ovulation protocols have been also used in cattle, and such protocols, which involve the use of progesterone-releasing devices and estradiol benzoate (EB), were found to yield more consistent results than Ovsynch protocols in *Bos indicus* cattle [10]. In addition, one study, in which a progestin (melengestrol acetate, MGA)-releasing device was implanted and estradiol and progesterone were administered on day 0, the device was withdrawn on day 6, PGF_2α_ was administered on day 7, a lower dose of estradiol was administered on day 8, and TAI was finally performed 28 h later, yielded a pregnancy rate of 60.7% [15]. Moreover, the inclusion of equine chorionic gonadotropin (eCG) in a progestin-based synchronization protocol when the progestin was withdrawn increased the pregnancy rate of suckled cows [16]. However, unfavorable conditions, such as the presence of the calf and inadequate nutrition, can negatively affect the success of progestin/estradiol-based TAI protocols by interfering with GnRH release, causing a reduction in LH pulsatility and impairing follicular development [17].

Other previous studies have shown that a presynchroni zation treatment (two injections of PGF_2α_, administered 14 days apart or an Ovsynch) prior to a breeding Ovsynch improve reproductive performance by increasing the proportion of cows that are at the most appropriate stage of estrus at the initiation of Ovsynch in dairy cows [18,19]. Of these, presynchronization with Ovsynch (Double-Ovsynch) has been shown to more reliably induce ovulation and improve the fertility of non-cycling cows [19]. However, studies of the Double-Ovsynch protocol in beef cattle have not been reported. We hypothesized that the use of the Double-Ovsynch protocol, fitting the timing of regular monthly veterinary visits, would promote the resumption of postpartum cyclicity and facilitate follicular growth and ovulation prior to the initiation of breeding Ovsynch in suckling beef cows, especially in those with prolonged postpartum anestrus, thereby improving the pregnancy rate associated with TAI. To test this hypothesis, we compared the efficacy of a modified Double-Ovsynch protocol, in which the interval of administration of hormones was adjusted according to the monthly herd visit schedule, with that of AI following estrus detection (AIED) with respect to the reproductive performance of Hanwoo cows.

## MATERIALS AND METHODS

### Animals

We studied 12 Hanwoo farms in Chungcheong Province, Korea, each of which housed 30 to 120 beef cattle. The cattle were maintained in indoor feedlots and fed concentrate, rice straw, minerals, and vitamins. Drinking water was provided *ad libitum*. All the feedlots were provided with fans and shade during the hot months (June to September). A total of 412 suckled Hanwoo cows (parity 2.2±0.1) were studied. All the experiments were performed according to the ethical guidelines of the Institutional Animal Care and Use Committee of Chungbuk National University, Korea (approval number CBNUA-1299-19-02).

### Study design

**Figure 1:**
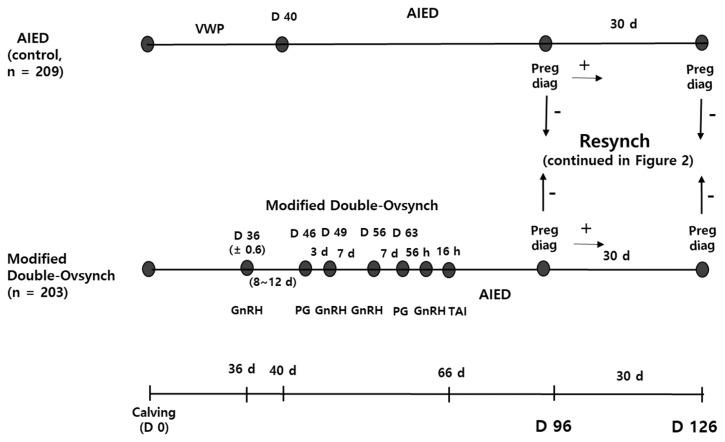
Study design with respect to the first AI following calving and pregnancy diagnosis. AIED, cows underwent AI following estrus detection; modified Double-Ovsynch, Ovsynch, during which the interval of administration of hormones was adjusted according to the herd visit schedule, followed by another Ovsynch 7 days later. AI, artificial insemination; AIED, artificial insemination following estrus detection; VWP, voluntary waiting period; D, day; Preg diag, pregnancy diagnosis; Resynch, resynchronization; GnRH, gonadotropin releasing hormone; PGF_2α_, prostaglandin F_2α_; TAI, timed artificial insemination.

Four hundred twelve Hanwoo cows were allocated to two treatment groups. The first group of cows was administered an injection of 10 μg of a GnRH analog, buserelin acetate (Gestar, Over, San Vicente, Argentina) on Day 36 (±0.6) (the calving date was designated as day 0), a PGF_2α_ analog, cloprostenol sodium (Estrumate, MSD Animal Health, Seoul, Korea) on Day 46 (8 to 12 days later; adjusted according to the herd visit schedule), and GnRH on Day 49; which was followed by Ovsynch, consisting of injections of GnRH on Day 56, PGF_2α_ on Day 63, and GnRH 56 h later, with TAI following a further 16 h later (modified Double-Ovsynch group, n = 203). The second group of cows underwent AIED (AIED group, n = 209) and was designated as the control group. Figure 1 shows the overall study design, including the hormone injections, AIs, and pregnancy diagnoses performed.

The following reproductive parameters were analyzed: i) the pregnancy per AI 30 and 60 days after the first AI; ii) the risk of pregnancy loss between 30 and 60 days of gestation following the first AI; iii) the likelihood of pregnancy by 150 days after calving; iv) the overall pregnancy per AI 60 days after the second and later AIs; and v) the mean number of inseminations per conception.

### Reproductive management

The voluntary waiting period between calving and the first AI was 40 days. All the cows underwent monthly reproductive health checks by veterinarians on the research team; these included an ultrasonographic examination of their ovarian structures and uterus (Tringa Linear VET Ultrasound Scanner, Esaote Pie Medical, Maastricht, The Netherlands). Pregnancy diagnosis was performed by transrectal ultrasonography 30 (±0.1) and 60 (±0.3) days after AI. Pregnancy loss was diagnosed when there was no embryonic heartbeat, there were no positive signs of pregnancy in a cow that had been previously diagnosed as being pregnant, or there were signs of embryo degeneration [20].

**Figure 2:**
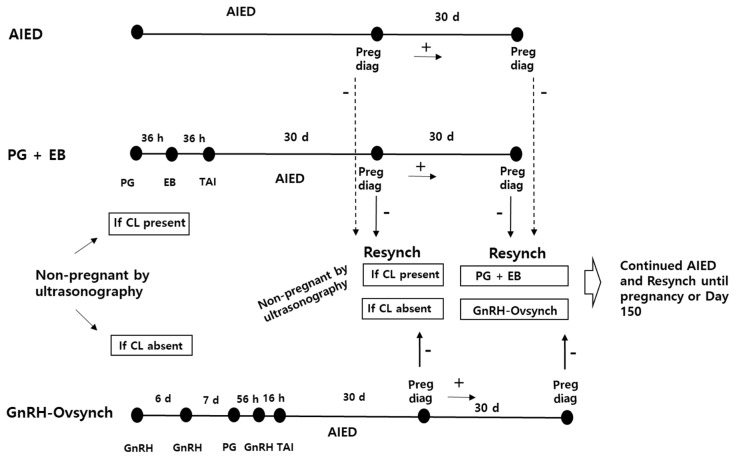
Re-inseminations following AIED or resynchronization programs in cows ultrasonographically confirmed as not being pregnant following the first or later AI, and pregnancy diagnoses. AIED, cows underwent AI following estrus detection; PG+EB, an injection of EB 36 h later and TAI a further 36 h later; GnRH-Ovsynch, an injection of GnRH, followed by Ovsynch 6 days later. AI, artificial insemination; AIED, artificial insemination following estrus detection; PGF_2α_, prostaglandin F_2α_; EB, estradiol benzoate; GnRH, gonadotropin releasing hormone; TAI, timed artificial insemination; d, day; Preg diag, pregnancy diagnosis; Resynch, resynchronization.

If cows in the modified Double-Ovsynch or AIED groups did not conceive following the first AI and exhibited estrus, they were inseminated (AIED) according to the am-pm rule. However, when cows were ultrasonographically confirmed as not being pregnant, those with a corpus luteum (CL) were resynchronized using an injection of PGF_2α_, followed by an injection of EB (SY Esrone, Samyang, Seoul, Korea) 36 h later and TAI a further 36 h later (PG+EB); and those without a CL were resynchronized using an injection of GnRH, followed by Ovsynch 6 days later (GnRH-Ovsynch). These resynchronization programs were continued until the cows became pregnant or 150 days after calving. The detailed resynchronization programs used for the second or later AI are shown in Figure 2. Reproductive performance data were collected for a minimum of 150 days postpartum.

### Statistical analyses

Results are expressed as means±standard error of the means. Statistical analyses were performed using SAS software (version 9.4; SAS Inst., Cary, NC, USA). For the statistical analyses, the season of AI was defined as spring (March to May), summer (June to August), autumn (September to November), or winter (December to February). The cows were also categorized according to herd size (<45 or ≥45 Hanwoo cows) and the number of days between calving and the first AI (<65 or ≥65 days). This interval for the modified Double-Ovsynch and AIED groups was compared using Student’s *t*-test.

The probability of pregnancy per AI following the first AI, the overall probability of pregnancy per AI after the second or later AI, and the probability of pregnancy loss between 30 and 60 days of gestation following the first AI were analyzed by logistic regression using the LOGISTIC procedure. To analyze the probabilities of pregnancy per AI following the first AI and of pregnancy loss following the first AI, we used logistic regression models that included treatment group (modified Double-Ovsynch and AIED), herd size, season of AI, the interval between calving and the first AI, and the interactions between these variables. To analyze the overall probability of pregnancy per AI following the second or later AI, the model included herd size, season of AI, the resynchronization program used (PG+EB, GnRH-Ovsynch, or AIED), the number of the AI (2, 3, or 4), and the interactions between these variables. Backward stepwise regression was used in all the models, and elimination was performed on the basis of the Wald statistic criterion when p>0.10. Odds ratios (ORs) and 95% confidence intervals (CIs) were determined using logistic regression.

A Cox’s proportional hazard model and the PHREG pro cedure were used to analyze the probability of pregnancy by 150 days postpartum. This yielded an estimate of the probability of a cow being pregnant at a given time. The time variable used in this model was the interval in days between calving and pregnancy. Cows that died, were sold, or were not pregnant by 150 days postpartum were not included in the analysis. The Cox model included the treatment group (modified Double-Ovsynch or AIED), herd size, the interval between calving and the first AI, and the interactions between these variables. The proportional hazard was determined on the basis of interactions between explanatory variables and time, and by evaluating Kaplan-Meier curves. The median and mean intervals between calving and conception were determined by survival analysis using the Kaplan-Meier model in the LIFETEST procedure within SAS software. A survival plot was generated using the Survival option within MedCalc (version 11.1; MedCalc Software, Mariakerke, Belgium).

Analysis of the number of inseminations required per conception was performed using a general linear model that included treatment group (modified Double-Ovsynch or AIED) and herd size as variables. Cows that died, were sold, or were not pregnant by 150 days postpartum were not included in the analyses. p≤0.05 was considered to represent statistical significance and 0.05<p<0.1 was considered to indicate a trend.

## RESULTS

The mean intervals between calving and the first AI after calving in the modified-Ovsynch and AIED groups did not significantly differ (66.1±0.7 and 63.9±1.4 days, respectively; p>0.1).

### Comparisons of the pregnancy per AI following the first AI, the overall pregnancy per AI following the second or later AI, and the rate of pregnancy loss between 30 and 60 days following the first AI

The pregnancy rates per AI 30 and 60 days after the first AI were 69.5% (141/203) and 68.5% (139/203), and 56.9% (119/209) and 56.5% (118/209) in the modified Double-Ovsynch and AIED groups, respectively. Table 1 shows the factors that affected the probability of pregnancy per AI following the first AI in each group. The probabilities of pregnancy per AI 30 (OR: 1.72, p<0.01) and 60 days (OR: 1.68, p<0.05) following the first AI were higher in the modified Double-Ovsynch group than in the AIED group. However, the herd size, season of AI, and the interval between calving and the first AI were not associated with the probability of pregnancy per AI (p>0.1).

The rates of pregnancy loss between 30 and 60 days of gestation following the first AI in the modified Double-Ovsynch and AIED groups were 1.4% (2/141) and 0.8% (1/119), respectively (p>0.1). In addition, none of the herd size, season of AI, or the interval between calving and the first AI affected these rates (p>0.1).

Table 2 shows the factors that affected the overall proba bility of pregnancy per AI 60 days after the second or later AI, determined using a logistic regression model. The use of resynchronization programs at the second or later AI (PG-EB for cows with a CL, GnRH-Ovsynch for cows without a CL, or AIED) and other variables, with the exception of the season of AI, did not affect the probability of pregnancy per AI after the second or later AI (p>0.1). Cows that were inseminated during the autumn were more likely (OR: 4.77, p<0.05), and those that were inseminated during the summer tended to be more likely (p<0.1), to become pregnant than those that were inseminated during the winter.

### Comparisons of the likelihood of pregnancy by 150 days postpartum and the mean number of inseminations per conception

**Figure 3:**
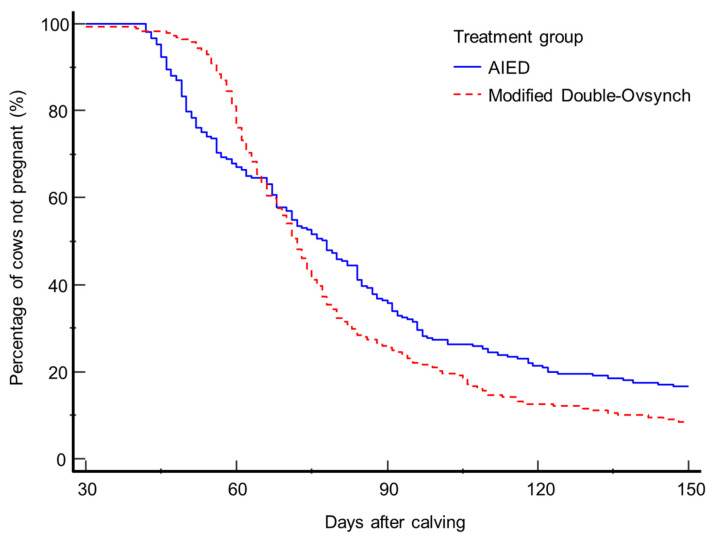
Survival curves generated using MedCalc software for the interval between calving and pregnancy for the modified Double-Ovsynch (n = 203) and AIED (n = 209) groups. The probability of pregnancy by 150 days postpartum was higher in the modified Double-Ovsynch group (hazard ratio: 1.28, p<0.05) than in the AIED group. The median and mean number of days between calving and pregnancy tended to be shorter (p<0.1) in the modified Double-Ovsynch group (72 and 81.9±2.1 days) than in the AIED group (78 and 85.9±2.5 days). AIED, artificial insemination following estrus detection.

Table 3 shows the factors affecting the likelihood of pregnancy by 150 days postpartum, determined using the PHREG procedure. Cows in the modified Double-Ovsynch group were more likely to have become pregnant by 150 days postpartum (hazard ratio [HR]: 1.28]) than those in the AIED group (p<0.05). The median and mean numbers of days between calving and pregnancy tended to be lower (p<0.1) in the modified Double-Ovsynch (72 and 81.9±2.1 days) than in the AIED (78 and 85.9±2.5 days) groups, as shown by the survival curves in Figure 3. In addition, the interval between calving and the first AI also affected the likelihood of a cow becoming pregnant. Cows that were inseminated <65 days after calving were more likely to become pregnant (HR: 2.12, p<0.01) than those inseminated ≥65 days after calving. However, cows in herds of ≥45 tended to be less likely to become pregnant (p<0.1) than those in herds of <45.

Table 4 shows the factors affecting the number of insemi nations required per conception. The least square mean number of inseminations required per conception was lower (p<0.05) in the modified Double-Ovsynch group (1.27±0.04) than in the AIED group (1.39±0.04). However, herd size did not affect the number of inseminations required per conception (p>0.1).

## DISCUSSION

In the present study, we aimed to determine whether a modified Double-Ovsynch protocol, in which the interval of administration of hormones was adjusted according to the herd visit schedule, could be efficiently implemented in Hanwoo herds and improve their reproductive performance over the use of AIED. We found that the modified Double-Ovsynch protocol improved certain reproductive parameters (higher probability of pregnancy per AI after the first AI and of a cow becoming pregnant by 150 days postpartum, and a lower mean number of inseminations required per conception) vs the use of AIED. Therefore, the modified Double-Ovsynch protocol could be easily used as a synchronization ovulation TAI protocol to improve reproductive performance in Hanwoo herds.

The present finding of a higher pregnancy per AI after the first AI in cows that underwent the modified Double-Ovsynch protocol (68.5%) than in those that underwent AIED (56.5%) suggests that the components of the modified Double-Ovsynch protocol, initiated as early as 36 days after calving, establish appropriate endocrine and reproductive conditions for pregnancy. The pregnancy per AI following the first AI in the Double-Ovsynch group in the present study was higher than those achieved in previous studies using only the Ovsynch or CO-Synch synchronization protocols (between 48% and 58%) [9,13,15]. Although the exact causes of the difference in reproductive performance between studies have not been determined, the addition of presynchronization (Ovsynch) in the present study would have promoted the resumption of postpartum cyclicity and facilitated follicular growth in cows with postpartum anestrus, contributing to favorable condition for ovulation synchronization during the breeding Ovsynch [19]. Consistent with this, a previous study showed that dairy cows which underwent Double-Ovsynch had a higher pregnancy per AI than those which underwent Ovsynch [21]. In another study, consisting of two experiments using a modified CO-Synch synchronization protocol, a 5-day CO-Synch, including the use of a CIDR, with TAI 72 h after CIDR removal, increased the pregnancy rate following TAI (65.5% and 80.0%) vs a 7-day CO-Synch, including the use of a CIDR, with TAI 60 h following CIDR removal (56.2% and 66.7%) [22].

As an alternative to the combined GnRH/PGF _2α_ synchronization ovulation protocols, many studies have used progestin-based TAI protocols based on estradiol and progesterone administration in an attempt to improve the reproductive performance of beef cows [15,23]. One study of progestin-based TAI protocols, including the administration of estradiol, progesterone, and PGF_2α_, achieved pregnancy per AI of between 48% and 61% in beef cows [15]. Furthermore, the addition of an eCG injection, to stimulate the growth of the dominant follicle and subsequent ovulation, at the time of progestin removal in progestin-based synchronization protocols, was associated with a pregnancy rate of 55.7% [16]. In a more recent study of suckled Nelore beef cows, an additional injection of GnRH 34 h after the removal of a progesterone insert as part of a modified estradiol/progesterone-based protocol resulted in a higher pregnancy per AI than in controls that did not receive GnRH (63.0% vs 50.4%) [24]. Taking all these findings together, in addition to the various well-designed modified Ovsynch, CO-Synch, and progestin-based TAI protocols described above, the modified Double-Ovsynch protocol, adjusted according to the herd visit schedule, can readily be implemented to increase the pregnancy per AI, possibly through an earlier resumption of cyclicity and better control of follicular growth and ovulation in suckling beef cows.

There was no difference in the rate of pregnancy loss be tween days 30 and 60 of gestation after the first AI between the modified Double-Ovsynch and AIED groups. The overall rate of 1.2% was lower than the 6.7% reported for Angus cows [25] and 5.0% for Nelore beef cows [26]. Although the timing of pregnancy loss (between 30 and 60 days after AI) in these studies was the same, it is unclear the difference in the rate of pregnancy loss can be explained by breed or other factors. We did not identify any factors that affected the overall pregnancy per AI after the second or later AI, with the exception of the season of AI. Our finding that cows inseminated during the winter had a lower rate of pregnancy following the second and later AI than those of cows inseminated during the summer or autumn may be explained by exposure to lower temperatures or inclement weather in the study area before becoming pregnant. Consistent with our finding, a previous study also showed that Japanese Black cattle that were inseminated during the winter and early spring had a lower pregnancy per AI than those that were inseminated during the summer and autumn [27]. They suggested that negative energy balance had a carryover effect on the quality of the developing oocytes due to an increased requirement for maintenance energy during the cold season before AI, impairing fertility [27].

Achieving a breeding target within a limited period of time is very important for beef herds. We found that modified Double-Ovsynch is more effective at achieving pregnancy in cows within a certain period of time (~150 days postpartum) than AIED. Furthermore, the higher likelihood of pregnancy by 150 days postpartum in the modified Double-Ovsynch group was associated with lower mean and median calving-to-pregnancy interval and fewer AIs required per conception than for the AIED group. Thus, financial gains owing to the reductions in effort and expense associated with the lower number of AIs and the shorter calving-to-pregnancy interval could be expected in cows subjected to the modified Double-Ovsynch protocol.

Our finding that a long interval between calving and the first AI is associated with a low pregnancy by 150 days after calving is consistent with the findings of a previous study [28]. The relationship between the long interval to the first AI and low pregnancy by 150 days after calving in the present study has not been clarified. But the delayed pregnancy may be related to the presence of postpartum diseases, such as retained placenta, endometritis, or metabolic disorder, impairing reproductive performance. The finding that cows in herds of ≥45 cows tended to be less likely to become pregnant than those in herds of <45 cows might reflect the greater need for labor or poorer management of treatment or nutrition than can be accomplished in larger herds. These findings suggest that the use of the modified Double-Ovsynch protocol in beef herds might be an effective means of regulating the timing of the first AI, shortening the interval to pregnancy after calving, and reducing the number of AIs required.

## IMPLICATIONS

Implementation of the modified Double-Ovsynch protocol, adjusted according to the herd visit schedule, improves reproductive performance (higher probabilities of pregnancy per AI after the first AI and of a cow becoming pregnant by 150 days postpartum, and fewer inseminations required per conception) vs AIED in cows. Therefore, the modified Double-Ovsynch protocol could be used as an efficient means of synchronizing ovulation as part of a TAI protocol by keeping the first AI as close as possible to the appropriate date following calving, improving the pregnancy rate after the first AI, and permitting short calving intervals in beef herds.

## Figures and Tables

**Table 1:** Factors affecting pregnancy per AI on the 30 and 60 days after the first AI in the modified Double-Ovsynch^1)^ and AIED^2)^ groups, identified using a logistic regression model

Parameter	Probability of pregnancy per AI (30 days after the first AI)				Probability of pregnancy per AI (60 days after the first AI)			
	
	Pregnancy per AI (n/n)	Odds ratio	95% CI	p-value	Pregnancy per AI (n/n)	Odds ratio	95% CI	p-value
Treatment group								
AIED	56.9% (119/209)	Reference			56.5% (118/209)	Reference		
Modified Double-Ovsynch	69.5% (141/203)	1.72	1.147–2.579	0.008	68.5% (139/203)	1.68	1.119–2.506	0.012
Herd size^3)^				0.511				0.526
Season of AI				0.484				0.501
Number of days to first AI^4)^				0.163				0.681

AI, artificial insemination; AIED, artificial insemination following estrus detection; CI, confidence interval; GnRH, gonadotropin releasing hormone; PGF_2α_, prostaglandin F_2α_.

1) Cows were administered GnRH on Day 36 (±0.6), PGF_2α_ on Day 46 (8 to 12 days later), GnRH on Day 49, GnRH on Day 56, PGF_2α_ on Day 63, and GnRH 56 h later, and were inseminated 16 h later.

2) Cows were artificially inseminated after estrus detection.

3) Herd size was categorized as <45 or ≥45 Hanwoo cows.

4) Cows were categorized according to the number of days between calving and the first AI (<65 or ≥65 days).

**Table 2:** Factors affecting the overall pregnancy per AI 60 on days after the second or later AI in Hanwoo cows

Parameter	Pregnancy per AI (n/n)	Odds ratio	95% CI	p-value
Season of AI				
Winter	40.0% (6/15)	Reference		
Spring	47.6% (20/42)	1.36	0.412–4.516	0.612
Summer	66.3% (63/95)	2.95	0.966–9.025	0.058
Autumn	76.1% (35/46)	4.77	1.388–16.416	0.013
Resynchronization program^1)^				0.202
AI number				0.179
Herd size^2)^				0.782

AI, artificial insemination; CI, confidence interval; TAI, timed artificial insemination; AIED, artificial insemination following estrus detection; GnRH, gonadotropin releasing hormone; PGF_2α_, prostaglandin F_2α_.

1) Includes AIED, PG+EB, and GnRH-Ovsynch. If cows did not conceive following the first or later AI and exhibited estrus, they were inseminated according to the am-pm rule (AIED); when cows were ultrasonographically confirmed as not being pregnant following the first or later AI, those with a CL were resynchronized using an injection of PGF_2α_, followed by an injection of estradiol benzoate (EB) 36 h later and TAI a further 36 h later (PG+EB); those without a CL were resynchronized using an injection of GnRH, followed by Ovsynch 6 days later (GnRH-Ovsynch). The resynchronization programs were continued until the cows became pregnant or 150 days after calving.

2) Herd size was categorized as <45 or ≥45 cows.

**Table 3:** Factors affecting the likelihood of pregnancy of Hanwoo cows by 150 days postpartum, analyzed using the PHREG procedure

Parameter	Hazard ratio	95% CI	p-value
Treatment group			
AIED^1)^ (n = 209)	Reference		
Modified Double-Ovsynch^2)^ (n = 203)	1.28	1.040–1.582	0.020
Number of days to first AI^3)^			
≥65 (n = 176)	Reference		
<65 (n = 236)	2.12	1.717–2.624	<0.01
Herd size^4)^			0.079

CI, confidence interval; AIED, artificial insemination following estrus detection; GnRH, gonadotropin releasing hormone; PGF_2α_, prostaglandin F_2α_; TAI, timed artificial insemination.

1) Cows were artificially inseminated after estrus detection.

2) Cows were administered GnRH on Day 36 (±0.6), PGF_2α_ on Day 46 (8–12 days later), GnRH on Day 49, GnRH on Day 56, PGF_2α_ on Day 63, and GnRH 56 h later, and were inseminated a further 16 h later. If cows did not conceive following the first or later AI and exhibited estrus, they were inseminated according to the am-pm rule (AIED). When cows were ultrasonographically confirmed as not being pregnant, those with a CL were resynchronized using an injection of PGF_2α_, followed by an injection of estradiol benzoate (EB) 36 h later and TAI a further 36 h later (PG+EB); and those without a CL were resynchronized using an injection of GnRH, followed by Ovsynch 6 days later (GnRH-Ovsynch). The resynchronization programs were continued until the cows became pregnant or 150 days after calving.

3) Cows were categorized according to the number of days between calving and the first AI (< 65 or ≥ 65 days).

4) Herd size was categorized as <45 or ≥45 cows.

**Table 4:** Least squares mean number of inseminations per conception

Parameter	No. of inseminations per conception	p-value
Treatment group		
AIED^1)^ (n = 174)	1.39±0.04	
Modified Double-Ovsynch^2)^ (n = 184)	1.27±0.04	0.0494
Herd size^3)^		0.7883

AIED, artificial insemination following estrus detection; GnRH, gonadotropin releasing hormone; PGF_2α_, prostaglandin F_2α_; TAI, timed artificial insemination.

1) Cows were artificially inseminated after estrus detection.

2) Cows were administered GnRH on Day 36 (±0.6), PGF_2α_ on Day 46 (8–12 days later), GnRH on Day 49, GnRH on Day 56, PGF_2α_ on Day 63, and GnRH 56 h later, and were inseminated a further 16 h later. If the cows did not conceive following the first or later AI and exhibited estrus, they were inseminated according to the am-pm rule (AIED). When the cows were ultrasonographically confirmed as not being pregnant, those with a CL were resynchronized using an injection of PGF_2α_, followed by an injection of estradiol benzoate (EB) 36 h later and TAI a further 36 h later (PG+EB); and those without a CL were resynchronized using an injection of GnRH, followed by Ovsynch 6 days later (GnRH-Ovsynch). The resynchronization programs were continued until the cows became pregnant or 150 days after calving.

3) Herd size was categorized as <45 or ≥45 cows.
